# Genome-Wide Analysis Unveils the Evolutionary Impact of Allopolyploidization on the 14-3-3 Gene Family in Rapeseed (*Brassica napus* L.)

**DOI:** 10.3390/genes16111305

**Published:** 2025-11-01

**Authors:** Shengxing Duan, Jing Wang

**Affiliations:** MARA Key Laboratory of Crop Ecophysiology and Farming System in the Middle Reaches of the Yangtze River, College of Plant Science and Technology, Huazhong Agricultural University, Wuhan 430070, China

**Keywords:** *Brassica*, 14-3-3, gene family, evolution, expression pattern

## Abstract

**Background**: Polyploidization drives the formation and evolution of angiosperms, profoundly reshaping genomic architecture and function. The 14-3-3 proteins (also known as G-box binding regulators, GRFs) are conserved signaling molecules involved in a range of physiological processes, including developmental signaling and stress responses. Elucidating the evolutionary trajectories of 14-3-3 genes in *Brassica napus* following allopolyploidization is critical for understanding polyploid crop evolution and developing molecular breeding strategies for improved stress resistance and yield. **Results**: In this study, forty-eight orthologous 14-3-3 genes were identified in the genome of *B. napus*, and twenty-two orthologous 14-3-3 genes were found in the genomes of both *Brassica rapa* and *Brassica oleracea*. Gene mapping analysis indicated that 14-3-3 genes were broadly distributed across all chromosomes; however, they exhibited significant heterogeneity. Phylogenetic tree construction revealed that 14-3-3 genes can be categorized into two groups: epsilon and non-epsilon genes. Gene structure analysis showed that most non-epsilon genes contain 3-4 exons, while most epsilon genes contain 5-7 exons. Collinearity analysis identified 36 orthologous gene pairs between the A (*B. rapa*) and C genomes (*B. oleracea*) but only 28 paralogous gene pairs within the A and C subgenomes of *B. napus*, indicating that some collinear 14-3-3 genes were lost during allopolyploidization. The *Ka/Ks* ratios (ratio of non-synonymous to synonymous substitution rate) of the 61 identified duplicated gene pairs were all less than 1, suggesting that these genes underwent purifying selection. Promoter analysis indicated that the average number of cis-acting elements in *B. napus* 14-3-3 genes was one more than in *B. rapa* and *B. oleracea*, implying that allopolyploidization increased the regulatory complexity of 14-3-3 genes. Tissue expression profiling demonstrated that the expression pattern of *GRF2* homologs was altered after allopolyploidization. **Conclusions**: By systematically investigating the copy number, genomic distribution, structure, evolutionary relationships, and expression patterns of 14-3-3 genes in *B. napus* and its progenitors, this study enhances our understanding of how allopolyploidization promotes gene family evolution.

## 1. Introduction

Polyploidization is a widespread evolutionary phenomenon in angiosperms [1,2,3,4] and plays a significant role in promoting speciation and evolutionary processes [1,2,3,4]. The formation of new polyploids is frequently accompanied by extensive genomic restructuring [5,6], which can lead to the subfunctionalization or neofunctionalization of homologous genes and concomitant alterations in their expression patterns [5,6]. Polyploidization substantially increases genomic complexity in plants and enhances trait diversity and environmental adaptability [7,8]. As a result, it is frequently utilized in crop domestication [8].

The 14-3-3 proteins were initially identified through separation from bovine brain proteins using column chromatography and electrophoretic mobility analysis [9]. As a component of nearly all eukaryotes, these proteins exhibit broad expression in diverse tissues [10]. 14-3-3 proteins were originally identified in the plant species *Arabidopsis thaliana* and became known as G-box binding regulators (GRFs) because their genes contain G-box elements [10]. Based on gene structure characteristics, 14-3-3 proteins can be classified into two distinct groups: epsilon and non-epsilon proteins [11]. These proteins play key regulatory roles as activators, repressors, or adaptors in numerous biological processes, such as hormone signaling [12,13,14,15,16], growth and development regulation [17,18,19,20], and stress responses [21,22,23,24,25,26,27]. Genome-wide identification of the 14-3-3 gene family has been conducted in various plant species, such as *A. thaliana* [10], rice [21], soybean [22], and alfalfa [28]. However, the evolutionary impact of allopolyploidization on this gene family remains poorly understood.

Globally, *Brassica napus* L. (AACC, 2n = 38) is an important oil crop in agriculture and economics [29]. This species originated approximately 7500 years ago from a natural hybridization event between the diploid progenitors *Brassica rapa* (AA, 2n = 20) and *Brassica oleracea* (CC, 2n = 18), which resulted in allotetraploidization [29,30]. This evolutionary event conferred considerable genetic diversity *B. napus*, inheriting stress resistance and high-yield traits from both parents, and established it as an ideal research model for evolutionary processes driven by allopolyploidization [30]. Recent advances in genome sequencing and high-quality assembly for *B. rapa* [31], *B. oleracea* [32], and *B. napus* [29] have provided deeper insights into its genetic architecture and breeding potential. In this study, we identified and compared 14-3-3 gene families across these three species. Comprehensive analyses were performed on their chromosomal locations, phylogenetic relationships, synteny, gene structures, selection pressures, cis-regulatory elements, and expression profiles to elucidate their evolutionary trajectories following allopolyploidization. Our findings provide important insights into the molecular evolution of 14-3-3 proteins and the identification of target genes to enhance stress resistance and yield in crops. Moreover, this study fills a critical gap in understanding how allopolyploidization shapes functional gene family evolution in *Brassica* crops and provides candidate genes for molecular breeding.

## 2. Materials and Methods

### 2.1. Identification of 14-3-3 Members

To identify putative 14-3-3 gene family members, the 13 known 14-3-3 protein sequences in *A. thaliana* [10] were used as queries for a BLASTP search against protein datasets of *B. napus* (v5.0), *B. rapa* (v1.5), and *B. oleracea* (v1.1) (available at http://brassicadb.cn, accessed on 26 August 2025) [33] using TBtools-II [34] with an E-value < 1E-5. The conserved domains of all candidate proteins were further verified using CDD (https://www.ncbi.nlm.nih.gov/Structure/cdd/wrpsb.cgi, accessed on 26 August 2025) [35], Pfam (http://pfam.xfam.org/, accessed on 26 August 2025) [36], and SMART (http://smart.embl.de/, accessed on 26 August 2025) [37]. The identified genes were subsequently renamed according to their homology with corresponding *A. thaliana* orthologs (Table S1).

### 2.2. Chromosomal Location, Gene Duplication, and Syntenic Analysis

The locations of the 14-3-3 genes were retrieved from the BRAD database. The chromosomal locations of the 14-3-3 genes were mapped using TBtools-II [34]. Gene pairs exhibiting >80% sequence length coverage and >80% identity according to BLASTN alignments were classified as duplicated genes [38]. The *Ka*/*Ks* ratios were calculated in TBtools-II via the Nei–Gojobori method, following codon-based MAFFT alignment. Sequences with gaps, stop codons, or *Ks* > 1 were excluded from the analysis. The time at which duplication events occurred was calculated as follows: *T* = *Ks*/2*λ* (*λ* = 1.5 × 10^−8^). Syntenic relationships were obtained from the BRAD database and visualized using Circos software (V0.69-9, available at http://circos.ca/) to illustrate the syntenic gene pairs [39].

### 2.3. Characterization of 14-3-3 Proteins

The ProtParam tool, available on the ExPASy server (https://www.expasy.org/, accessed on 26 August 2025) [40], was employed to analyze various physicochemical parameters of the 14-3-3 proteins, such as the molecular weight (MW), grand average of hydropathicity (GRAVY), instability index (II), and isoelectric point (pI).

### 2.4. Phylogenetic Tree Construction and Analysis

To reconstruct the evolutionary relationships among the 14-3-3 proteins, sequences from *A. thaliana*, *B. napus*, *B. rapa*, and *B. oleracea* were first aligned with ClustalW, implemented in MEGA7 [41]. From this alignment, a neighbor-joining phylogenetic tree was generated using the same software, with branch support assessed through 1000 bootstrap replications. Then, this tree was visualized and annotated with iTOL (https://ngphylogeny.fr/, accessed on 26 August 2025) [42].

### 2.5. Gene Structure and Motif Identification

Gene structures and conserved motifs were visualized using TBtools-II [34] and the MEME suite [43], respectively.

### 2.6. Prediction of Cis-Acting Elements

Cis-acting elements in the 14-3-3 genes’ 2000 bp upstream sequences at the transcription start site (TSS) were predicted using the PlantCARE database (https://bioinformatics.psb.ugent.be/webtools/plantcare/html/, accessed on 26 August 2025) [44].

### 2.7. Analysis of Gene Expression

RNA-seq data were obtained from a previous study [45]. The raw reads, comprising three biological replicates for each of the four major tissues (flowers, stems, leaves, and siliques), are publicly available in the NCBI database under accession numbers SRR7816633 to SRR7816668. Clean sequence reads were aligned to the reference genome using HISAT2 (v2.1.0) [46] under default settings. Following this alignment, only uniquely mapped reads were considered for subsequent quantification. The gene expression was quantified with the software featureCounts (v1.6.1) [47] by counting reads mapped to the reference genomes. Subsequently, the read counts for each gene were normalized to TPM (transcripts per million). Visualization of the expression profiling data was performed using Microsoft Excel.

## 3. Results

### 3.1. Identification of 14-3-3 Genes

**Figure 1:**
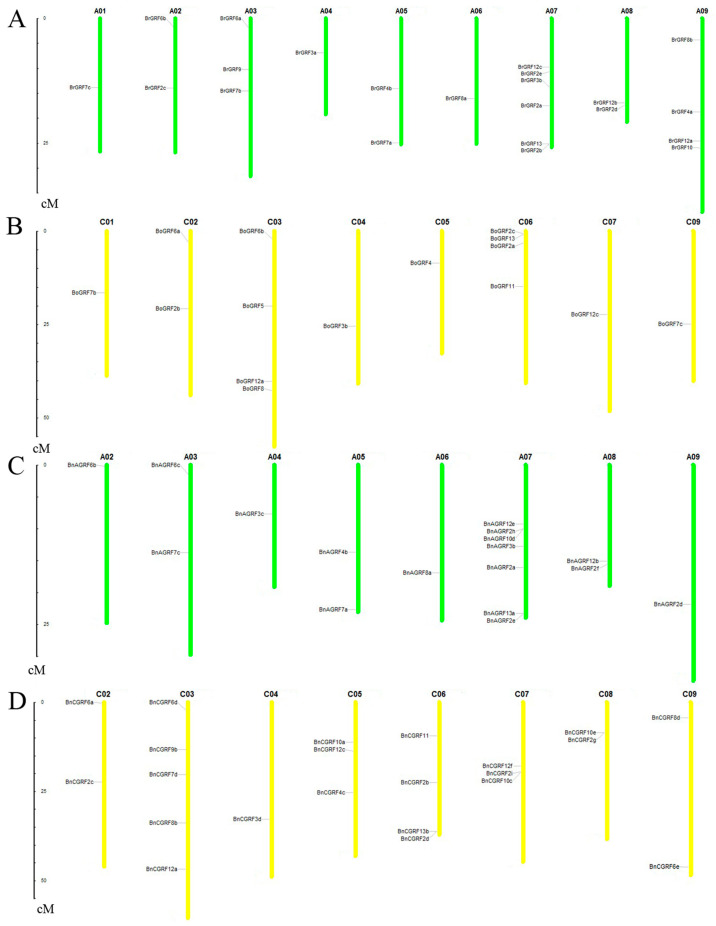
Chromosomal localization of 14-3-3 genes in *B. rapa* (**A**), *B. oleracea* (**B**), An of *B. napus* (**C**), and Cn of *B. napus* (**D**). Genes located in unassembled scaffolds are not shown. The number of chromosomes is marked at the top of each, and the scale on the left is given in megabases (Mb).

To identify members of the 14-3-3 gene family, the *A. thaliana* 14-3-3 protein sequences were used as queries to perform a BLASTP search against the genomes of *B. napus*, *B. rapa*, and *B. oleracea*, respectively. After screening for conserved domains, a total of 48 candidate proteins were identified in *B. napus*, and 22 each were identified in *B. rapa* and *B. oleracea*. All identified genes were systematically renamed according to their homology with *A. thaliana* genes (Table S1). Chromosome localization analysis indicated that the 14-3-3 genes were unevenly distributed across nearly all chromosomes in *B. napus*, *B. rapa*, and *B. oleracea* (Figure 1), suggesting an extended 14-3-3 gene family in these genomes compared to *Arabidopsis*.

### 3.2. Phylogenetic Analysis of 14-3-3 Gene Families

**Figure 2:**
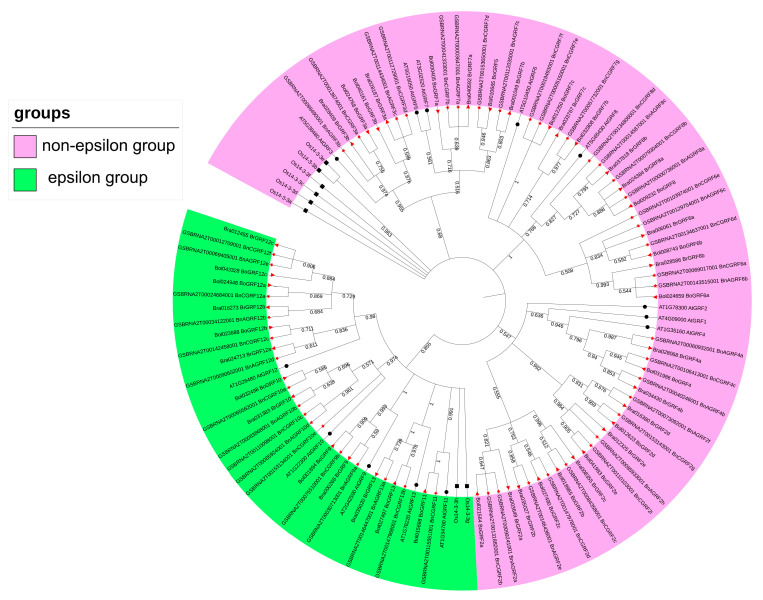
Phylogenetic trees of 14-3-3 genes from *A. thaliana*, *O*. *sativa*, *B. rapa*, *B. oleracea*, and *B. napus*.

To elucidate the phylogenetic and evolutionary relationships between 14-3 and 3 genes, a phylogenetic tree was constructed using protein sequences from five plant species: *A. thaliana*, *Oryza sativa*, *B. napus*, *B. rapa*, and *B. oleracea*. *A. thaliana* and *O. sativa* were designated as the outgroup to ensure accurate phylogenetic inference. The resulting tree demonstrated that the 14-3-3 genes were divided into two groups: epsilon and non-epsilon groups (Figure 2). This classification aligns with previously established groupings in *A. thaliana* [10,11] and rice [21], supporting the high conservation of the 14-3-3 gene family classification across plant species.

### 3.3. Gene Structure and Motif Analysis

**Figure 3:**
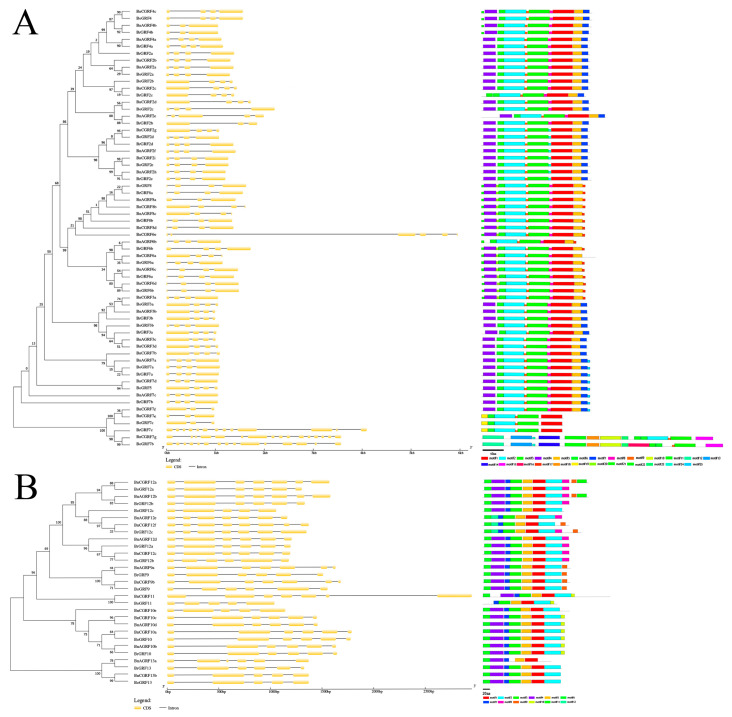
Gene structures and protein domains of 14-3-3 genes. The neighbor-joining phylogenetic trees, gene structures, and motifs of the non-epsilon group (**A**) and epsilon group (**B**). Yellow boxes indicate exons, and gray lines represent introns. Different motifs are distinguished by colored boxes.

Phylogenetic trees of the 14-3-3 gene families were generated using the neighbor-joining method. The exon–intron structures and conserved motifs were subsequently analyzed separately for the non-epsilon group (Figure 3A) and epsilon group (Figure 3B) in *B. napus*, *B. rapa*, and *B. oleracea*, respectively. Most genes contained 3 or 4 exons in the non-epsilon group (Figure 3A) and 5-7 exons (Figure 3B) in the epsilon group. Genes clustered on the same phylogenetic branch exhibited similar exon–intron architectures, implying evolutionary conservation. The motif analysis revealed 25 motifs in the non-epsilon group (Figure 3A) and 12 motifs in the epsilon group (Figure 3B). Notably, genes on the same branch shared similar motif compositions, although the number and type of motifs differed considerably between the two groups.

### 3.4. Synteny and Duplicated Gene Analysis of 14-3-3 Genes

**Figure 4:**
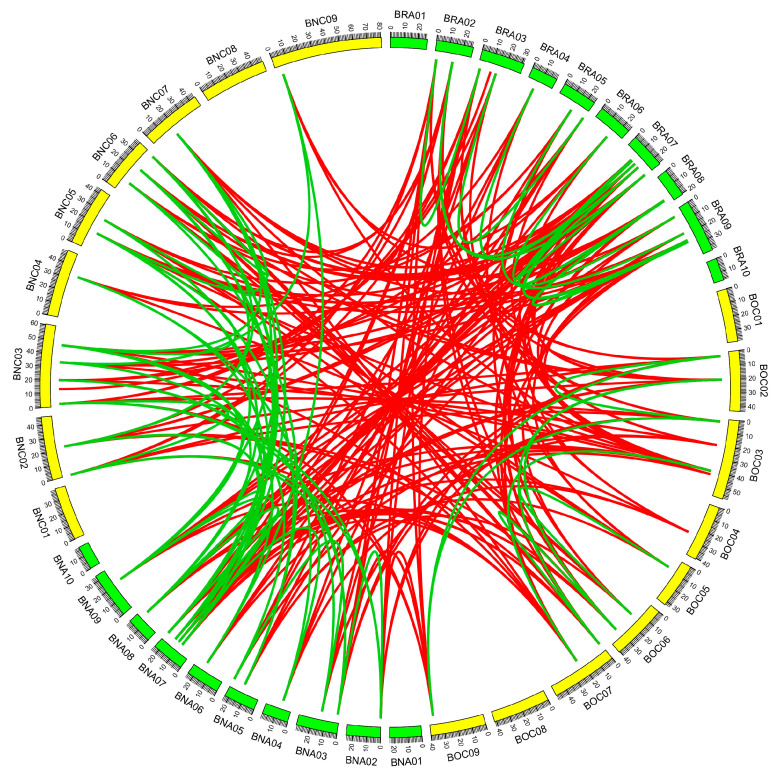
Collinearity analysis of 14-3-3 genes from *B. rapa*, *B. oleracea*, and *B. napus*. BRA01-10 and BOC01-09 represent chromosomes in *B. rapa* and *B. oleracea*, respectively. BNA01-10 and BNC01-09 represent chromosomes in the An and Cn subgenomes in *B. napus*, respectively. All identified 14-3-3 genes were mapped onto corresponding chromosomes. Red lines link the orthologs, and green lines link the paralogs.

The synteny analysis of 14-3-3 genes was performed using genomic location information from *B. napus* (A_n_ and C_n_ subgenomes), *B. rapa* (A_r_), and *B. oleracea* (C_r_). As a result, 67 paralogous gene pairs (within A_n_ and C_n_) and 172 orthologous gene pairs (between A_n_ and A_r_ and C_n_ and C_r_) (Figure 4) were identified, suggesting the potential loss of some syntenic 14-3-3 genes during polyploidization. Additionally, 12, 10, and 39 duplicated gene pairs were detected in the genomes of *B. rapa*, *B. oleracea*, and *B. napus*, respectively (Table 1). These duplication events were estimated to have occurred 1.74–16.72 million years ago (MYA). The *Ka*/*Ks* analysis revealed that all 61 duplicated gene pairs exhibited *Ka*/*Ks* ratios less than 1, suggesting that they had undergone purifying selection. Furthermore, all duplicated gene pairs except *BnAGRF13a*-*BnCGRF13b* were under strong purifying selection (*Ka*/*Ks* < 0.5). These results imply that the duplicated 14-3-3 gene pairs in *B. napus* and its two diploid progenitors were subject to purifying selection pressure following duplication.

### 3.5. Prediction of Physicochemical Properties of 14-3-3 Proteins

The molecular weight (MW) of 14-3-3 proteins in *B. napus* and its two diploid progenitors varied from 22,690 Da (*BoGRF7c*, *BnCGRF7e*, *BnCGRF7f*) to 65,584 Da (*BnCGRF7g*) (Table S1). On average, the MW of *B. napus* 14-3-3 proteins (30,190 Da) was slightly lower than its diploid progenitors (30,635 Da), which was attributed to polyploidization. The isoelectric point (pI) of 14-3-3 proteins ranged from 4.61 to 9.66. Aside from *BoGRF7b/c* and *BnCGRF7e/f/g*, all 14-3-3 proteins were classified as acidic (with pI < 7). Furthermore, all 14-3-3 proteins except *BrGRF12c*, *BrGRF13*, *BnCGRF10e,* and *BnAGRF13a* exhibited considerable instability indices greater than 40. Conversely, the aliphatic index of all 14-3-3 proteins exceeded 70, suggesting higher thermal stability. Additionally, the grand average of hydropathicity (GRAVY) was negative for all 14-3-3 proteins, consistent with their hydrophilicity.

### 3.6. Analysis of Cis-Acting Elements in 14-3-3 Genes

**Figure 5:**
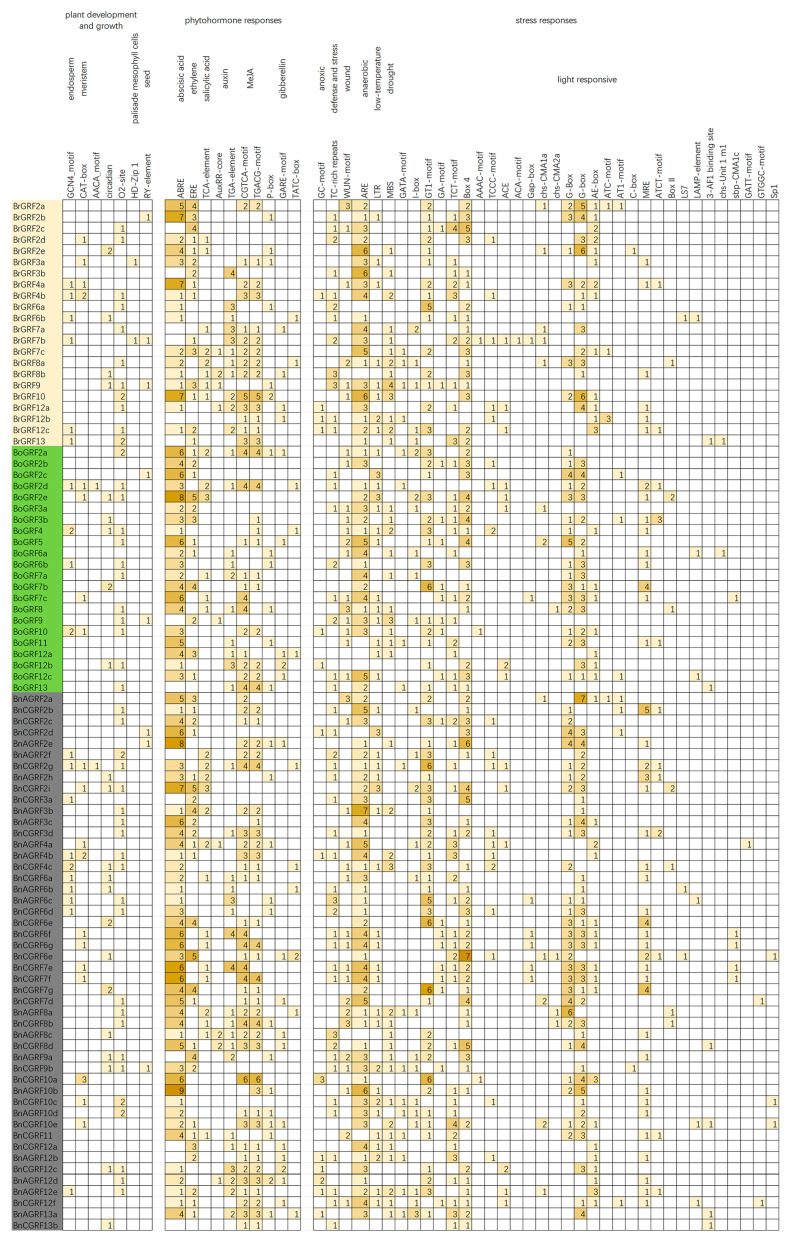
Cis-acting element analysis of 14-3-3 gene promoters from *B. rapa (Br)*, *B. oleracea (Bo)*, and *B. napus (Bn)*.

To investigate whether polyploidization influences the potential regulatory functions of 14-3-3 genes, we identified cis-acting elements within the 2000 bp promoter regions upstream of their transcription start sites. Three functional categories of cis-elements were examined: plant development and growth, phytohormone response, and stress responses (Figure 5). On average, the promoter of *B. napus* 14-3-3 genes contained 26 cis-acting elements, 1 more than those in *B. rapa* and *B. oleracea*, which was attributed to polyploidization. Among the plant development and growth-related elements, the GCN4_motif (associated with endosperm expression) and CAT-box (involved in meristem activity) were detected. The 14-3-3 gene promoters were also enriched in phytohormone response-related elements; many genes contained over six ABREs (abscisic acid-responsive elements) and more than four EREs (ethylene-responsive elements), indicating a potential role in hormone regulation. Additionally, cis-elements with light responsiveness such as Box-4 and G-box were abundant in these gene promoters, suggesting their role in light-mediated regulation. Interestingly, multiple ARE elements linked to anaerobic stress induction were also identified. The prevalence of these elements suggests the involvement of 14-3-3 genes in stress response mechanisms.

### 3.7. Expression Patterns of 14-3-3 Genes in Different Tissues

**Figure 6:**
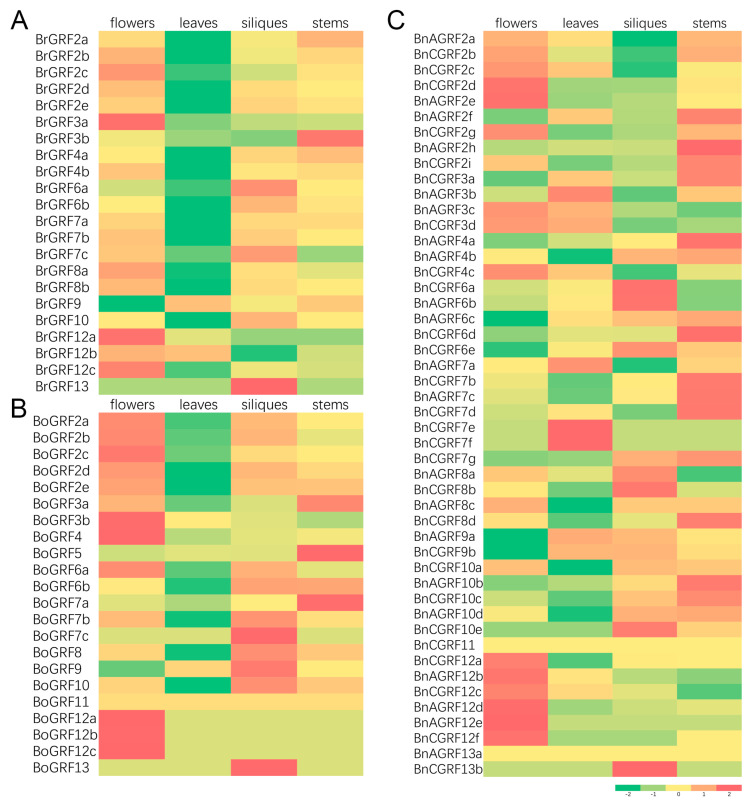
Expression of 14-3-3 genes in flowers, leaves, siliques, and stems of *B. rapa* (**A**), *B. oleracea* (**B**), and *B. napus* (**C**). Red represents high expression, while green indicates low expression.

To further investigate the expression and potential biological functions of all identified 14-3-3 genes, their expression profiles across four tissues (leaves, stems, flowers, and siliques) were analyzed using previously published RNA-seq data [45]. Aside from *BoGRF11* in *B. oleracea* and *BnAGRF13a* in *B. napus*, which were not expressed in any of the four tissues, all other genes showed detectable expression. As illustrated in Figure 6, *BrGRF6b*, *BoGRF6a*, and *BnAGRF6b* showed notably high expression in flowers and siliques, implying functionally conserved roles. Overall, the average expression level of 14-3-3 genes was higher in flowers, siliques, and stems than in leaves in both *B. rapa* and *B. oleracea*. In contrast, *B. napus* showed higher expression in flowers and stems compared to siliques and leaves (Figure 6). In *B. rapa* and *B. oleracea*, the expression of several genes, including *GRF2* homologs, was especially low in leaves but significantly higher (1.7- to 2.0-fold) in flowers, siliques, and stems. In *B. napus*, however, the expression of these homologs was lower in siliques than in other tissues, indicating that polyploidization may have altered the expression patterns.

## 4. Discussion

Polyploidization is a significant evolutionary driving force of angiosperm diversity, promoting genome reorganization, functional differentiation, and adaptive innovation [48]. In newly formed allotetraploid crops such as *B*. *napus* [29], it is important to understand how polyploidy reshapes gene families to decipher the molecular basis of its advantages and unlock genetic potential for crop improvement. This study focuses on the 14-3-3 gene family, a conserved signaling hub, to dissect the multifaceted effects of allopolyploidization. By integrating genomic, structural, regulatory, and expression data, we reveal that polyploidy drives gene family evolution through the coordinated interplay of gene copy number modulation, regulatory network integration, functional conservation, and adaptive divergence. These mechanisms are broadly applicable to fundamental gene families and thus provide potential targets for improving polyploid crops.

### 4.1. Allopolyploidization as a Driver of Gene Family Expansion

In this study, the increased 14-3-3 gene copy number in *B*. *napus* resulted from the combined effects of multiple duplication mechanisms. The genus *Brassica* shares a common ancestor with *A*. *thaliana* [29]. Following phylogenetic divergence, the *Brassica* lineage underwent a whole-genome triplication event and subsequent genomic rearrangements, ultimately leading to the differentiation of *B. rapa* and *B. oleracea* [31,32]. Subsequently, *B. napus* originated from interspecific hybridization between *B. rapa* and *B. oleracea*, followed by natural chromosome doubling [29]. Polyploidization, or whole-genome duplication, significantly drives gene family expansion, while tandem duplication and segmental duplication at the gene level are also common drivers of copy number evolution and family expansion [48]. Notably, all 14-3-3 homologous genes identified in this study were classified as products of segmental duplication, with no tandem duplication events detected, indicating that this is the dominant mechanism for the expansion of this gene family in *B. napus*. Furthermore, the *Ka*/*Ks* ratios of duplicated gene pairs were all less than 1, indicating strong purifying selection, which is attributed to the functional integrity of these genes across species while permitting subtle functional innovations [49,50].

In summary, allopolyploidization is the primary factor driving the numerical expansion of the 14-3-3 gene family in *B. napus*, further reinforced by frequent segmental duplications. Similar duplication patterns have also been observed in other multigene families in *B. napus*, such as the *WOX* [51] and *EIL* [52] families, reflecting a certain degree of commonality in its genomic evolutionary pathways.

### 4.2. Polyploidization Drives Regulatory Pattern Diversification

Polyploidization not only influences gene copy number but also profoundly reshapes gene regulatory networks (Figure 5). Promoter analysis revealed that *B. napus* possesses, on average, one more cis-acting element per 14-3-3 gene compared to its diploid progenitors (Figure 5). This increase suggests that polyploidization may enhance the regulatory complexity of these genes, potentially facilitating their involvement in diverse biological processes. Elements such as ABRE, ERE, G-box, and ARE were abundant, underscoring the roles of 14-3-3 genes in abiotic stress adaptation and developmental regulation [45,53]. This enrichment and diversification of regulatory elements likely stem from the integration and innovation of regulatory modules from two distinct ancestral genomes during polyploid formation. Although the coding sequences of the 14-3-3 genes themselves were conserved under purifying selection, this implies that their expression “switches” became more refined and diverse. Such diversification in regulatory patterns likely provides *B. napus* with broader flexibility in regulating expression, enabling it to more effectively connect endogenous hormonal signals to external environmental stresses, thereby enhancing the environmental adaptability and plasticity of polyploid plants. This represents an important advantage of polyploids at the regulatory level [54].

### 4.3. Polyploidization Drives Structural and Biochemical Adaptation of 14-3-3 Proteins

In addition to changes in regulation and expression, the structural and physicochemical properties of 14-3-3 proteins also exhibit notable evolutionary trends. The reduced average molecular weight of *B. napus* compared to its progenitors may reflect structural optimization, potentially influencing protein interactions or stability (Table S1). The prevalence of acidic isoelectric points and large instability indices (above 40) in most 14-3-3 proteins suggests that they are generally unstable but hydrophilic, which may facilitate flexible signaling mediation in dynamic cellular environments. The high aliphatic index indicates robust thermal stability, a feature likely conserved to ensure functionality under stress conditions [55]. These biochemical characteristics underline the adaptive evolution of 14-3-3 proteins in balancing functional versatility with structural resilience.

### 4.4. Polyploidization Drives Diversification of Expression Patterns

The ultimate function of genes is reflected in their expression patterns. Tissue-specific expression profiling revealed that allopolyploidization has modified the expression patterns of 14-3-3 genes in *B. napus* (Figure 6). While genes in diploid species showed high expression in flowers, siliques, and stems, their homologs in *B. napus* exhibited reduced expression in siliques, indicating that polyploidization may have rewired their transcriptional regulatory networks. Notably, certain genes, such as *BrGRF6b*, *BoGRF6a*, and *BnAGRF6b*, maintained high expression in reproductive tissues across species, suggesting functional conservation. This rewiring of expression patterns is likely closely associated with the changes in regulatory sequences. This diversification in expression patterns provides a foundation for functional innovation in polyploid plants. In *B. napus*, this may enable optimized growth and developmental programs and potential trait advantages absent in its progenitors. This is crucial for its successful adaptation and domestication [45,56].

### 4.5. Evolutionary Implications and Future Perspectives

This study elucidates how allopolyploidization has shaped the evolution of the 14-3-3 gene family in *B. napus*, primarily through driving gene family expansion and reorganization, enhancing regulatory complexity, and diversifying expression patterns. Together, these mechanisms have conferred novel flexibility to the regulation and expression of this otherwise functionally conserved gene family in the context of polyploids. Our findings further provide broader insights into the evolutionary strategies employed by polyploid plants. The coexistence of gene loss, purifying selection, regulatory diversification, and expression reprogramming illustrates the multifaceted impact of allopolyploidization on gene family evolution. In *B. napus*, the 14-3-3 genes have evolved through a balanced process of genetic conservation and regulatory innovation, enhancing adaptive capacity without compromising core functions. Future research on the functional validation of specific genes, such as those with altered expression or unique regulatory elements, will be crucial for elucidating the mechanistic links between genetic evolution and phenotypic adaptation in polyploid crops. Moreover, the candidate genes identified, particularly those associated with stress and hormone responses, hold the potential to improve crop resilience and productivity through molecular breeding.

## Figures and Tables

**Table 1:** Estimated *Ka/Ks* ratios of duplicated 14-3-3 gene pairs in *B. napus* and its diploid progenitors.

Duplicated Gene Pairs			*Ka*	*Ks*	*Ka*/*Ks*	Duplication Type	Types of Selection	Time (MYA)
*BrGRF2a*	vs.	*BrGRF2b*	0.0208	0.3966	0.052446	Segmental	Purify selection	13.22
*BrGRF2a*	vs.	*BrGRF2c*	0.0215	0.3535	0.06082	Segmental	Purify selection	11.78333
*BrGRF2b*	vs.	*BrGRF2c*	0.0316	0.3394	0.093105	Segmental	Purify selection	11.31333
*BrGRF2d*	vs.	*BrGRF2e*	0.0495	0.4794	0.103254	Segmental	Purify selection	15.98
*BrGRF3a*	vs.	*BrGRF3b*	0.0245	0.4011	0.061082	Segmental	Purify selection	13.37
*BrGRF4a*	vs.	*BrGRF4b*	0.034	0.3114	0.109184	Segmental	Purify selection	10.38
*BrGRF6a*	vs.	*BrGRF6b*	0.0559	0.3705	0.150877	Segmental	Purify selection	12.35
*BrGRF7a*	vs.	*BrGRF7b*	0.0175	0.3308	0.052902	Segmental	Purify selection	11.02667
*BrGRF8a*	vs.	*BrGRF8b*	0.0177	0.4204	0.042103	Segmental	Purify selection	14.01333
*BrGRF12a*	vs.	*BrGRF12b*	0.0265	0.3298	0.080352	Segmental	Purify selection	10.99333
*BrGRF12a*	vs.	*BrGRF12c*	0.0468	0.2898	0.161491	Segmental	Purify selection	9.66
*BrGRF12b*	vs.	*BrGRF12c*	0.0327	0.3236	0.101051	Segmental	Purify selection	10.78667
*BoGRF2a*	vs.	*BoGRF2b*	0.0176	0.3443	0.051118	Segmental	Purify selection	11.47667
*BoGRF2a*	vs.	*BoGRF2c*	0.0229	0.4037	0.056725	Segmental	Purify selection	13.45667
*BoGRF2b*	vs.	*BoGRF2c*	0.0321	0.3123	0.102786	Segmental	Purify selection	10.41
*BoGRF2d*	vs.	*BoGRF2e*	0.0523	0.5017	0.104246	Segmental	Purify selection	16.72333
*BoGRF3a*	vs.	*BoGRF3b*	0.0244	0.3522	0.069279	Segmental	Purify selection	11.74
*BoGRF5*	vs.	*BoGRF7a*	0.0192	0.3223	0.059572	Segmental	Purify selection	10.74333
*BoGRF6a*	vs.	*BoGRF6b*	0.0302	0.422	0.071564	Segmental	Purify selection	14.06667
*BoGRF12a*	vs.	*BoGRF12b*	0.0262	0.3152	0.083122	Segmental	Purify selection	10.50667
*BoGRF12a*	vs.	*BoGRF12c*	0.0641	0.3298	0.19436	Segmental	Purify selection	10.99333
*BoGRF12b*	vs.	*BoGRF12c*	0.0457	0.3263	0.140055	Segmental	Purify selection	10.87667
*BnAGRF2a*	vs.	*BnCGRF2b*	0	0.0575	0	Segmental	Purify selection	1.916667
*BnAGRF2a*	vs.	*BnCGRF2c*	0.0241	0.3105	0.077617	Segmental	Purify selection	10.35
*BnAGRF2a*	vs.	*BnCGRF2d*	0.0194	0.3585	0.054114	Segmental	Purify selection	11.95
*BnAGRF2a*	vs.	*BnAGRF2e*	0.0208	0.3882	0.053581	Segmental	Purify selection	12.94
*BnCGRF2b*	vs.	*BnCGRF2c*	0.0241	0.3447	0.069916	Segmental	Purify selection	11.49
*BnCGRF2b*	vs.	*BnCGRF2d*	0.0523	0.4536	0.1153	Segmental	Purify selection	15.12
*BnCGRF2b*	vs.	*BnAGRF2e*	0.0208	0.4163	0.049964	Segmental	Purify selection	13.87667
*BnCGRF2c*	vs.	*BnCGRF2d*	0.0339	0.2875	0.117913	Segmental	Purify selection	9.583333
*BnCGRF2c*	vs.	*BnAGRF2e*	0.0392	0.313	0.12524	Segmental	Purify selection	10.43333
*BnCGRF2d*	vs.	*BnAGRF2e*	0.005	0.1125	0.044444	Segmental	Purify selection	3.75
*BnAGRF3b*	vs.	*BnAGRF3c*	0.021	0.4534	0.046317	Segmental	Purify selection	15.11333
*BnAGRF3b*	vs.	*BnCGRF3d*	0.021	0.4095	0.051282	Segmental	Purify selection	13.65
*BnAGRF3c*	vs.	*BnCGRF3d*	0.0017	0.1109	0.015329	Segmental	Purify selection	3.696667
*BnAGRF4b*	vs.	*BnCGRF4c*	0.0117	0.0932	0.125536	Segmental	Purify selection	3.106667
*BnCGRF6a*	vs.	*BnAGRF6c*	0.0399	0.4429	0.090088	Segmental	Purify selection	14.76333
*BnCGRF6a*	vs.	*BnCGRF6d*	0.0321	0.4324	0.074237	Segmental	Purify selection	14.41333
*BnAGRF6c*	vs.	*BnCGRF6d*	0.0099	0.1354	0.073117	Segmental	Purify selection	4.513333
*BnAGRF7a*	vs.	*BnAGRF7c*	0.0175	0.3219	0.054365	Segmental	Purify selection	10.73
*BnAGRF7a*	vs.	*BnCGRF7d*	0.0175	0.3133	0.055857	Segmental	Purify selection	10.44333
*BnAGRF7c*	vs.	*BnCGRF7d*	0.0083	0.102	0.081373	Segmental	Purify selection	3.4
*BnAGRF8a*	vs.	*BnCGRF8b*	0.0018	0.1617	0.011132	Segmental	Purify selection	5.39
*BnAGRF8a*	vs.	*BnCGRF8d*	0.0186	0.4227	0.044003	Segmental	Purify selection	14.09
*BnCGRF8b*	vs.	*BnCGRF8d*	0.0162	0.3483	0.046512	Segmental	Purify selection	11.61
*BnCGRF12a*	vs.	*BnAGRF12b*	0.0079	0.0522	0.151341	Segmental	Purify selection	1.74
*BnCGRF12a*	vs.	*BnCGRF12c*	0.0276	0.3337	0.082709	Segmental	Purify selection	11.12333
*BnCGRF12a*	vs.	*BnAGRF12d*	0.0244	0.3345	0.072945	Segmental	Purify selection	11.15
*BnCGRF12a*	vs.	*BnAGRF12e*	0.0389	0.3673	0.105908	Segmental	Purify selection	12.24333
*BnCGRF12a*	vs.	*BnCGRF12f*	0.0825	0.4056	0.203402	Segmental	Purify selection	13.52
*BnAGRF12b*	vs.	*BnCGRF12c*	0.0267	0.3046	0.087656	Segmental	Purify selection	10.15333
*BnAGRF12b*	vs.	*BnAGRF12d*	0.0235	0.3053	0.076973	Segmental	Purify selection	10.17667
*BnAGRF12b*	vs.	*BnAGRF12e*	0.0352	0.3192	0.110276	Segmental	Purify selection	10.64
*BnAGRF12b*	vs.	*BnCGRF12f*	0.079	0.3765	0.209827	Segmental	Purify selection	12.55
*BnCGRF12c*	vs.	*BnAGRF12d*	0.0064	0.1598	0.04005	Segmental	Purify selection	5.326667
*BnCGRF12c*	vs.	*BnAGRF12e*	0.0496	0.2853	0.173852	Segmental	Purify selection	9.51
*BnCGRF12c*	vs.	*BnCGRF12f*	0.0629	0.3035	0.207249	Segmental	Purify selection	10.11667
*BnAGRF12d*	vs.	*BnAGRF12e*	0.048	0.2504	0.191693	Segmental	Purify selection	8.346667
*BnAGRF12d*	vs.	*BnCGRF12f*	0.0632	0.2956	0.213802	Segmental	Purify selection	9.853333
*BnAGRF12e*	vs.	*BnCGRF12f*	0.0049	0.1385	0.035379	Segmental	Purify selection	4.616667
*BnAGRF13a*	vs.	*BnCGRF13b*	0.0387	0.0713	0.542777	Segmental	Purify selection	2.376667

## Data Availability

The RNA-seq data came from a previous study [45].

## Supplementary Materials

The following supporting information can be downloaded at https://www.mdpi.com/article/10.3390/genes16111305/s1, Table S1: The predicated protein information for 14-3-3 genes from *B. napus*, *B. rapa*, and *B. oleracea*.

## Abbreviations

The following abbreviations are used in this manuscript:

*B. napus*	*Brassica napus*
*B. rapa*	*Brassica rapa*
*B. oleracea*	*Brassica oleracea*
*A. thaliana*	*Arabidopsis thaliana*
GRFs	G-box binding regulators
MW	Molecular weight
GRAVY	Grand average of hydropathicity
II	Instability index
pI	Isoelectric point
MYA	Million years ago
